# SCiMS: Sex Calling in Metagenomic Sequences

**DOI:** 10.64898/2026.02.17.705110

**Published:** 2026-02-18

**Authors:** Hanh N. Tran, Kobie J. Kirven, Emily R. Davenport

**Affiliations:** 1 Department of Biology, Pennsylvania State University, University Park, PA; 2 Huck Institutes of the Life Sciences, Pennsylvania State University, University Park, PA; 3 Department of Chemistry, Pennsylvania State University, University Park, PA

**Keywords:** Metagenomics, sex inference, host sex, microbiome

## Abstract

**Background:**

Host sex is a critical determinant of microbial community structure, influenced by hormonal profiles, physiology, and sex-stratified behaviors. Despite its importance, sex metadata is frequently missing or mislabeled in microbiome studies. Existing genomic sex-calling tools often fail in low-host-biomass samples (e.g., stool) because they require high read depths to achieve reliability.

**Results:**

Here, we present SCiMS (Sex Calling in Metagenomic Sequences), a bioinformatic tool that leverages host-derived DNA within metagenomic datasets to accurately predict host sex, even at low host coverage. SCiMS uses sex-chromosome read density ratios within a Bayesian classifier to provide high-accuracy sex calls. In simulations, SCiMS achieves >85% accuracy with as few as 450 host reads. When applied to 1,339 samples from the Human Microbiome Project, SCiMS outperforms existing tools, showing higher accuracy and more balanced precision-recall tradeoffs across body sites. SCiMS also generalizes effectively to non-human hosts, achieving 100% accuracy in a murine dataset and outperforming alternatives in a chicken dataset with a ZW sex determination system.

**Conclusions:**

SCiMS provides an accurate, scalable, and cross-species generalizable solution for host sex classification in metagenomic datasets, even when host DNA is minimal. By enabling the recovery of missing sex metadata, it serves as a quality-control tool for ensuring the integrity of analyses in microbiome research. SCiMS is freely available at http://github.com/davenport-lab/SCiMS.

## Introduction

Host sex plays a significant role in shaping microbiome composition and function. A multitude of studies have reported sex-specific differences in gut and other microbiota [1–6]. For example, a large human cohort study found that females harbored higher relative abundances of Firmicutes and Actinobacteria, whereas males showed enrichment of Bacteroidetes [2,6]. As a result, recognizing host sex as a key variable in microbiome research is critical to ensure more accurate interpretation of biological patterns in both clinical and ecological contexts.

Despite its importance, a large portion of microbiome-related sequencing projects are missing host sex information. A brief survey of the NCBI Sequence Read Archive in May 2025 returned over 500,000 human-derived shotgun metagenomic samples. Of these samples, over 90% are missing host sex information. In addition, paucity of host sex metadata extends beyond human studies. Our survey of the AnimalAssociatedMetagenomeDB [7] showed that host sex information was missing from 97.2% of chicken (*Gallus gallus*), 85.3% of cattle (*Bos taurus*), and 100% of pigs (*Sus domesticus*) entries. Even for the common model organism, the house mouse (*Mus musculus*), 97.6% of samples failed to include host sex information. This shortfall could simply be due to researchers not recording sex at the time of sampling, missing entries, or working with samples where an organism’s sex cannot be easily determined. Consequently, those aiming to analyze sex-specific patterns or simply control for sex as a confounding factor often find that large portions of otherwise valuable sequence data cannot be used.

Because host-derived reads are routinely present in metagenomic datasets, host sex can be inferred directly from sequence data alone even when sex metadata are missing [8]. Several genomic approaches infer sex from whole-genome sequencing data by comparing the ratios of host reads that map to the sex chromosomes or sex chromosomes versus autosomes, including BeXY [9], Rx [10], and Ry [11]. BeXY classifies sex-linked scaffolds by modeling differences in chromosomal ploidy (copy number) across samples using sequencing read counts [9]. Rx calculates the average ratio of reads mapping to X chromosome relative to reads mapping to each autosome [10]. Ry calculates the fraction of reads mapping to Y chromosome relative to reads mapping to both X and Y chromosomes [11]. However, they require moderately high depth of host reads to reliably call sex. This poses a challenge for metagenomic samples where host reads can be sparse, particularly in microbe-rich samples such as tongue or stool where host-derived reads typically comprise only a small fraction of total sequences, only 19% and 1%, respectively [12]. While the potential of calling host sex from metagenomic reads has been demonstrated [8, 18], no tools currently exist that enable metagenomic sex calling approaches to be easily incorporated into metagenomic analysis pipelines. Moreover, systematic benchmarking has yet to be performed to assess the accuracy of existing methods at the low host read depths commonly observed in host-derived metagenomic datasets. Together, these limitations motivate the need for methods that reliably infer host sex information from metagenomic sequencing data alone, even when host read coverage is minimal.

Here we introduce SCiMS—Sex Calling in Metagenomic Sequences—a command-line tool that determines host sex directly from shotgun metagenomic data, even when only a few hundred host reads are present. SCiMS employs a Bayesian probabilistic model to infer sex based on host read mapping information in organisms with heterogametic sex determination systems (XY or ZW). Compared with existing tools, SCiMS delivers higher accuracy at much lower host read depths and outputs a simple metadata text file that can be incorporated into any metagenomic pipeline. This allows users to include sex classification as a routine quality control check or as an explicit covariate in downstream ecological and clinical analyses even when sex metadata is not available.

## Materials and methods

### SCiMS workflow and sex-calling algorithm

SCiMS predicts host sex using the predictable differences in read coverage between sex chromosomes and autosomes. In species with heterogametic sex determination systems (XY or ZW), autosomes are present in two copies in both sexes while the sex chromosomes vary by sex. In XY systems, females carry two copies of the X chromosome, while males carry one X and one Y. As a result, coverage on the X and Y chromosomes varies systematically with sex (and vice versa in ZW systems). SCiMS quantifies these differences using two normalized coverage metrics: Rx modified from Mittnik et al. [10] and Ry [11]

$$Rx=\frac{1}{n}\sum_{i=1}^{n}\;\left(\frac{X\;\mathrm{coverage}\;}{i^{\mathrm{th}}\;\mathrm{autosome}\;\mathrm{coverage}}\right);Ry=\frac{Y\;\mathrm{reads}\;}{X\;\mathrm{reads}\;+\;Y\;\mathrm{reads}}$$

In practice, deviations from these canonical values often arise due to sparse host DNA and stochastic variation in low-coverage regions. To robustly model this variability across sequencing depths, SCiMS uses a Gaussian kernel density estimation (KDE) framework. To build training reference data for the model, we generated 24,000 simulated read-pair samples from male and female GRCh38-based reference genomes across twelve read depth targets ranging from 150 to 1,000,000 reads. SCiMS uses these simulations to construct two KDE models, one for each sex, that estimate the likelihood of observing any (Rx, Ry) pair under male or female assumptions. For each new metagenomic sample, SCiMS calculates (Rx, Ry), evaluates its likelihood under both KDE models, and then combines those likelihoods with equal prior probabilities to compute posterior probabilities for each sex. If the posterior probability for the more likely sex is ≥0.80 (default threshold), SCiMS reports that classification. Otherwise, it assigns the sample as “uncertain”. Users may adjust this threshold to suit their precision-recall preferences.

### Comparative method performance

To benchmark SCiMS, we directly compared its performance against three existing sex inference tools originally developed for genomic data: BeXY, Rx, and Ry. We applied all methods to the same idxstats input files to ensure a consistent basis for comparison. We evaluated each method using standard classification metrics: accuracy, precision, recall, and F1 score, calculated separately for each sex as following:

$$Precision=\frac{TP}{TP\;+\;FP};\;Recall\;=\frac{TP}{TP\;+\;FN};\;F1\;score\;=2\frac{\;precision\;\times \;recall\;}{\;precision\;+\;recall\;}$$

where TP (true positives) denotes correct predictions, FP (false positive) refers to incorrect predictions, and FN (false negatives) represents cases where the method is abstained (i.e., uncertain calls).

### Simulated dataset

Metagenomic samples often contain a low proportion of host-derived reads. To evaluate how well SCiMS perform under such conditions, we simulated sequencing data across a wide range of host read depths. We generated a male and a female human reference genome from the genome assembly GRCh38.p14 using a custom script. The male reference included a full diploid genome (autosomes plus both X and Y chromosomes), while the female reference retained all autosomes along with two copies of the X chromosome. From each reference genome, we simulated 10 million paired-end reads using wgsim v.0.3.1-r13 [14], applying default sequencing-error parameters. Reads were aligned back to the GRCh38 assembly using Bowtie2 v.2.5.2 [15]. To simulate varying levels of host DNA abundance, we downsampled the full-depth BAM files without replacement to 9 target read depths: 150, 250, 350, 450, 1,000, 5,000, 10,000, 100,000, and 1,000,000 reads. For each depth and sex, we generated 1,000 replicates using the downsample.py script. Chromosome-level alignment statistics were extracted using SAMtools idxstats [14] and used as input for four sex-calling tools: SCiMS, BeXY [9], Rx [10], and Ry [11]. For SCiMS evaluation, we compared the results of SCiMS and other methods to the provided ground truth labels for each simulated sample.

### Real metagenomic datasets

To determine whether simulated performance translated to real metagenomic data, we applied SCiMS to three real metagenomic datasets: Human, mouse, and chicken (Table 1).

### Data preprocessing

For simulated samples, we directly aligned the reads to human GRCh38.p14 assembly using Bowtie2 v.2.5.2 [15]. For real metagenomic datasets, we trimmed adapters and filtered reads for quality using fastp v.0.23.4 [20] with default parameters. For all datasets, we used only samples with at least 100 host reads. We then mapped the filtered reads to the corresponding host reference genomes (human GRCh38.p14, mouse GRCm39, and chicken GCA_027557775.1) using Bowtie2 v.2.5.2 [15]. After alignment, we removed potential PCR duplicates with the MarkDuplicates function in Picard Tools v.3.1.1 (http://broadinstitute.github.io/picard/). We further refined the alignments by retaining only uniquely mapped reads with mapping quality (MAPQ) ≥ 30, using SAMtools v.1.19 [14]. To avoid confounding effects from pseudoautosomal regions (PARs), which are present on both X and Y chromosomes and share high sequence similarity [21], we explicitly excluded these regions from the human dataset using BEDtools v.2.31.0 [22]. Finally, we used SAMtools’ function idxstats to extract the number of reads mapped to each chromosome or scaffold, generating per-sample read count profiles that served as the primary input for SCiMS.

## Results

### Overview of SCiMS

We developed SCiMS, a command-line tool designed to recover host sex metadata directly from metagenomic alignments. Unlike methods relying on fixed thresholds for sexing, SCiMS uses a Bayesian framework to quantify the probability of sex assignment. Specifically, it calculates the read density of the homogametic chromosome (e.g. X) relative to that of autosomes, and the read counts of heterogametic chromosome (e.g. Y) relative to the total sex chromosome pool of each sample. These observed ratios are then evaluated against sex-specific empirical distributions generated via Kernel Density Estimation (KDE) from training data. By integrating these likelihoods with a uniform prior probability, SCiMS computes a joint posterior probability distribution for each sex, assigning a final classification only when the posterior probability exceeds a user-defined threshold. For more details on the SCiMS model, see Supplementary methods.

**Fig. 1.**
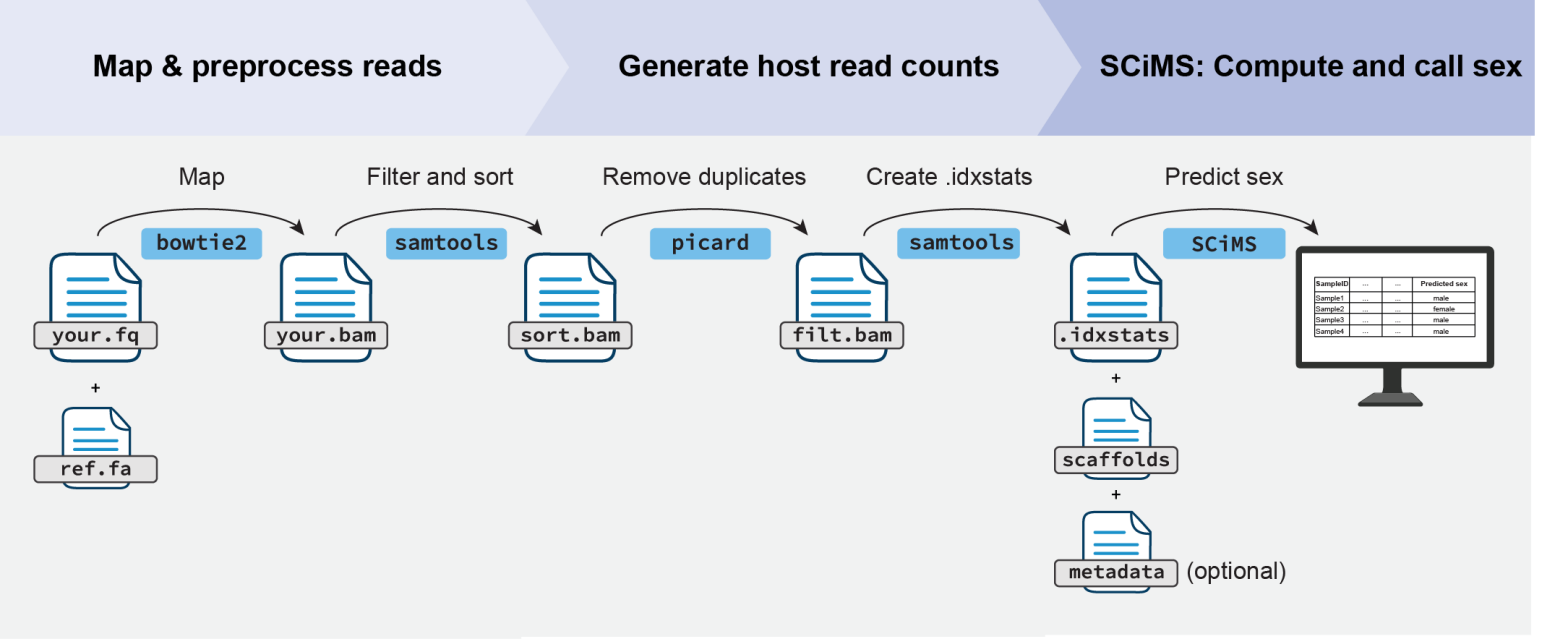
Suggested workflow. Shotgun metagenomic reads (your.fq) should first be aligned to the host reference genome (ref.fa) with a mapping tool (such as Bowtie2) to produce a BAM file (your.bam). The alignment should then be sorted and filtered for mapping quality with SAMtools (sort.bam), after which PCR duplicates should be removed with Picard or similar (filt.bam). SCiMS takes as input the idxstats file generated by SAMtools (.idxstats) which supplies read counts per chromosome, a text list of chromosome or scaffold identifiers from the host reference genome, and optionally, a metadata table. SCiMS computes Rx and Ry ratios and returns a probabilistic sex call displayed in a tabular summary and amended to the metadata table if supplied.

The recommended workflow begins with preprocessing of raw sequencing reads (Fig. 1). First, users align reads to a host reference genome using an aligner such as Bowtie214. To improve classification accuracy, we recommend removing PCR duplicates with Picard, then sorting and filtering the BAM files using SAMtools to retain only high-quality, uniquely mapped reads (MAPQ ≥ 30). When annotations are available, filtering out pseudoautosomal regions (PARS) helps avoid coverage biases from regions shared between sex chromosomes [15]. In cases where PARs are not annotated, SCiMS still performs well, as shown in simulated data (Supplementary Fig. 1). After cleaning, users generate an. idxstats file from the aligned reads. This file summarizes the number of reads mapped to each chromosome or scaffold and serves as input for SCiMS. From this, SCiMS calculates the Rx and Ry metrics and classifies sex based on a probabilistic model built on a training dataset.

SCiMS requires two main inputs: (1) an ‘.idxstats’ file and (2) a text file listing chromosome or scaffold IDs (Fig. 1). Optionally, users can adjust the classification threshold for Bayesian posterior probabilities based on their desired sensitivity versus specificity, with the default set to 0.80. We selected this default threshold based on a precision-recall analysis across a range of posterior cutoffs (0.50–0.99). Among these, a threshold of 0.80 provided a balanced trade-off between precision and recall across read depths, while maintaining a low uncertainty rate and high overall accuracy (Supplementary Fig. 2). Users wishing for higher specificity should select a threshold closer to 0.99, whereas users wishing for higher sensitivity can lower it from the 0.8 default value. Additionally, SCiMS supports both XY and ZW sex determination systems through the ‘–sex-system’ option, making it applicable to a wide range of organisms with heterogametic sex determination systems. A complete tutorial and implementation guide is available at (https://github.com/davenport-lab/SCiMS).

### SCiMS correctly assigns sex when host reads are scarce

**Fig. 2.**
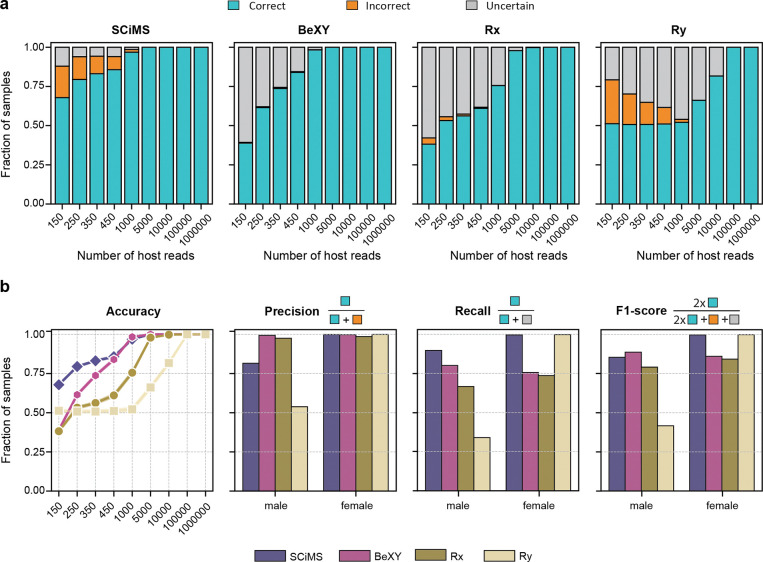
SCiMS outperforms existing sex-calling methods across all simulated host-read depths. **a)** SCiMS achieves the highest proportion of correctly classified samples compared to BeXY, Rx, and Ry in simulated datasets at the default posterior cutoff of 0.8. Each bar represents 1000 male and 1000 female simulated samples at the indicated host-read depth (150–1,000,000 reads). **b).** SCiMS maintains a balanced performance for both male and female samples, whereas BeXY and Rx trade recall for precision, particularly in females, and Ry skews strongly toward female calls, leading to the lowest male F1 score across all method.

To evaluate performance across varying levels of host read depths, we benchmarked SCiMS on 18,000 simulated samples spanning a broad range of sequencing reads (150 to 1,000,000 reads) (Fig. 2a). We compared SCiMS against BeXY, Rx, and Ry. While all methods achieved near-perfect classification at high host read counts (>10,000 reads), SCiMS demonstrated higher sensitivity in low coverage samples. Even at the lowest read count tested (150 host reads), SCiMS correctly recovered over 67% of samples, representing a 1.7-fold increase in data recovery compared to BeXY (38.9%), Rx (38.2%). This improvement stems from SCiMS’ ability to integrate signals from X, Y, and autosomal chromosomes into a probabilistic framework that explicitly models distribution under sparse coverage. Notably, SCiMS maintained the lowest uncertainty rate across all depths (Fig. 2a), providing confident calls where other methods failed. As read depth increased, accuracy improved rapidly, exceeding 95% accuracy at just 1,000 host reads.

We further stratified performance by sex to assess potential classification bias (Fig. 2b). SCiMS showed a balanced performance profile, giving high F1 scores for both males (0.82) and females (0.99). This symmetry confirms that SCiMS uses information from both sex chromosomes to classify sex without systemically favoring one class. In contrast, BeXY and Rx showed a sharp trade-off between precision and recall. While their predictions were often correct when made (i.e., high precision), they frequently abstained or misclassified low-coverage samples, especially for females, leading to lower overall F1 scores. Finally, Ry, which relies solely on Y-linked reads, systematically underperformed on male samples (recall = 0.339, F1 = 0.416), reflecting its tendency to misclassify low-coverage males as females when Y reads were too sparse to be detected.

Together, these results on simulated data highlight SCiMS’ ability to assign host sex with high accuracy and confidence, even in ultra-low host read conditions. These results establish SCiMS as an effective tool for recovering host sex signals from sparse data.

### SCiMS achieves accurate and balanced host sex classification across human metagenomic samples

**Fig. 3.**
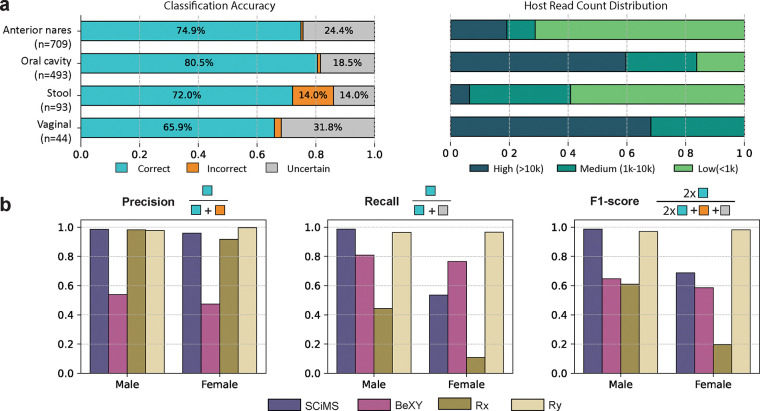
SCiMS accurately classifies host sex from metagenomic data across multiple human body sites. **a)** SCiMS’ classification results and host read distribution for 1,339 Human Microbiome Project samples from anterior nares (n=709), oral cavity (n=493), stool (n=93), and vaginal samples (n=44). SCiMS attains high accuracy (65.9 – 80.5%) even in stool samples where host DNA is typically less than 1%. **b)** Sex-stratified precision, recall, and F1-scores pooled across all body sites for SCiMS, BeXY, Rx, and Ry. SCiMS maintains a relatively balanced performance for males and females, similar to Ry. BeXY shows worst performance with F-1 scores < 0.5 for both sexes while Rx achieves high precision but low recall in both sexes, resulting in reduced F1-scores.

To evaluate SCiMS on real-world data, we applied it to 1,339 human metagenomic samples from the Human Microbiome Project [12], all of which included ground truth sex labels (Supplementary Table 4). The samples represented four body sites: anterior nares (n=709; 41% female), oral cavity (n=493; 58% female), stool (n=93; 61% female), and vaginal swabs (n=44, 100% female). SCiMS demonstrated consistent reliability across these diverse environments, achieving high precision in host sites such as the anterior nares (>99.0%) and oral cavity (98.8%). Beyond high precision, SCiMS maintained robust sensitivity across varying levels of host biomass (Fig. 3a). In the oral cavity, SCiMS correctly recovered sex information for 80.5% of samples. Even in challenging contexts like stool samples, where host DNA typically constitutes less than 1% in the sample [12], SCiMS successfully resolved 72.0% of samples.

In addition to high overall accuracy, SCiMS maintained low misclassification rates in anterior nares, the oral cavity, and vaginal samples, from 0.7 to 2.3% (Fig. 3a). Stool samples, in contrast, showed the highest misclassification rate among sites at 14.0%. This reduced accuracy likely reflects the low proportion of host DNA in fecal material, which can obscure sex specific signals and increase the likelihood of classification errors. Specifically, over 93% of stool samples consist of less than 10,000 host reads whereas samples from the other body sites such as oral cavity and vaginal swabs have over 60% and 68% of samples exceeding 10,000 host reads per sample, respectively (Fig. 3b). Correspondingly, accuracy generally improves as host read depth increases across all sites (Supplementary Fig. 4).

When compared to other tools, SCiMS outperformed both BeXY and Rx across all body sites (Supplementary Fig. 3). BeXY’s accuracy ranged from 30.8% (oral samples) to 68.2% (vaginal samples) (Supplementary Table 3). Rx performed even more poorly, with classification accuracy as low as 17.1% in anterior nares and 18.2% in vaginal samples. In contrast, Ry performed surprisingly well in anterior nares, oral, and vaginal samples (accuracy >95%). This result is likely driven by the high specificity of the Ry metric in high-biomass samples, where host DNA is relatively abundant due to mucosal or epithelial shedding as commonly observed in oral, nasal, and vaginal metagenomic samples [12, 16, 17]. In these contexts, the abundance of X-chromosome reads creates a large denominator in the Ry calculation (Methods). This effectively ensures that those samples with high X-chromosome reads are correctly classified as female (Ry ≈ 0) rather than being misclassified as males due to noise. Consistent with this explanation, anterior nares, oral and vaginal samples had average mapped host read depths of 89,000, 144,000, and 552,000. However, in stool samples where host DNA is sparse (average depth = 3,800), we observed reduced accuracy (59.1%) in Ry.

We further assessed method performance by evaluating sex-specific precision, recall, and F1 scores (Fig. 3b and Supplementary Table 4). SCiMS demonstrated high balanced performance across male and female classification. For males, SCiMS achieved a high F1 score of 0.98, reflecting both high precision (0.99) and recall (0.97). While the F1 score for females was lower (0.68), this was driven primarily by reduced recall (0.52) rather than precision (0.96). Importantly, the decrease in recall was largely attributable to SCiMS withholding predictions in uncertain cases, rather than assigning incorrect classifications. This pattern suggests that SCiMS maintains strong positive predictive power across sexes while prioritizing precision by abstaining from confident sex calls when the input data are ambiguous or insufficient.

Compared to SCiMS, BeXY and Rx showed lower overall performance across most evaluation metrics. Specifically, BeXY yielded F1 scores of 0.51 (male) and 0.44 (female) with moderate precision but notably low recall. Rx showed a sharp drop in female performance (F1= 0.19), due to extremely low recall (0.11), suggesting the method often abstains or misclassifies females in real-world data. Together, these results highlight SCiMS’ ability to integrate both Rx and Ry information to make robust sex calls, even under conditions where other methods return inconclusive results or make errors.

### SCiMS accurately assigns host sex in non-human metagenomic data

**Fig. 4.**
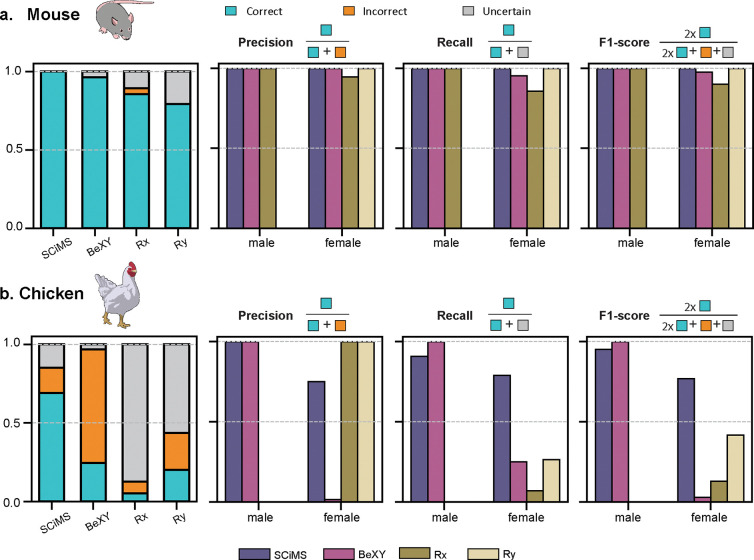
SCiMS shows better performance compared to other tools for non-human metagenomic datasets. **a)** Performance comparison across all four methods on mouse fecal samples (n=111). **b)** Equivalent comparison on chicken cecal samples (n=94). For each dataset, the first panel shows the fraction of samples that were correctly classified, misclassified, or left uncertain by each method. The remaining panels summarize precision, recall, and F1 score for each sex class, quantifying the accuracy, sensitivity, and balance of classification performance across methods. SCiMS demonstrates the highest accuracy and balanced performance across sex classes in both datasets, particularly in chicken where other methods show increased misclassification or sex bias. Mouse and chicken illustrations from NIAID NIH BioArt Source https://bioart.niaid.nih.gov/bioart/280 [34] and https://bioart.niaid.nih.gov/bioart/131 [35].

We next evaluated SCiMS on two non-human datasets: mouse (*Mus musculus*) [18] and chicken (*Gallus gallus*) [19–21], representing an alternative mammal with an XY and birds with a ZW sex determination system, respectively (Fig. 4, Table 1, and Supplementary Table 5). In the mouse cecal dataset, SCiMS achieved perfect performance, correctly classifying all 111 samples with no incorrect or uncertain calls. BeXY, Rx, and Ry also performed well (96.4%, 84.6%, and 78.3% accuracy, respectively), likely reflecting the high proportion of host DNA recovered from murine cecal samples (average host read depth = 3,250,000 reads). However, SCiMS stood out for its precision and recall across both male and female mice, achieving balanced F1 scores near 1.0 (Fig. 4a).

We then tested SCiMS in an organism with a ZW system in which females are the heterogametic sex: chicken. Compared to mammals, avian species present a more difficult setting for sex inference due to their poorly assembled W chromosome and a greater divergence between Z and W chromosome [22, 23]. Despite the challenging setting, SCiMS achieved 69.1% accuracy, outperforming all other methods. BeXY and Ry reached only 24.5% and 20.2% accuracy, respectively, while Rx was only at 5.3% (Fig. 4b). These discrepancies highlight the limitations of existing tools, which are tailored to XY systems and do not account for the distinct coverage signatures in ZW genomes. SCiMS, in contrast, supports both XY and ZW systems and can model differences in sex chromosome dosage accordingly. Although SCiMS was not trained specifically on avian genomes, the tool achieved the highest recall of all methods (male: 0.91, female: 0.75). In contrast, BeXY misclassified over 70% of samples while Rx and Ry demonstrated poor recall across both sexes (Fig. 4b). Overall, SCiMS demonstrates its generalizability to ZW systems, providing a valuable resource for predicting sex in diverse non-model organisms.

## Discussion

SCiMS is a new tool that accurately determines host sex from metagenomic samples, including those with low host read coverage. This ability to function effectively at low host sequencing depths fills a critical gap in metagenomic analysis, because host reads can be sparse in metagenomic data depending on the site of sampling and unevenly distributed across the genome. With a Bayesian probabilistic framework that integrates depth-normalized signals from sex chromosomes and autosomes, SCiMS enhances reliability of sex calls beyond other available tools by incorporating prior information about the likelihood of each sex and updating these probabilities as new data is observed. This approach allows SCiMS to weigh both the observed data and prior knowledge and expectations about the likelihood of each sex in order to make an accurate call of host sex. Thus, SCiMS is robust because it can handle uneven coverage and sparse host data better than existing methods, making it more effective in the low-host read scenarios often encountered with metagenomic samples.

The implications of SCiMS’s performance are significant, and it has a wide range of use cases across fields. First, in biomedical research metagenomic data is increasingly being collected for studying the microbiome’s role in health and disease. Accurate sex determination can be crucial for understanding sex-specific disease manifestations and tailoring treatments accordingly. We have benchmarked SCiMS on two of the most commonly used organisms in biomedical research to demonstrate its performance: Humans and mice. Second, SCiMS will be useful for studies of wildlife microbiomes, where host sex information may not be available. This could occur in cases where fecal samples are collected from the field without a behavioral observation accompanying collection or from species that demonstrate low sexual dimorphism, making it challenging to sex an organism from a distance or without tranquilizing the animal. The repository for SCiMS includes instructions on how to run SCiMS on new, user-defined genomes so long as the organism has a heterogametic sex determination system. Finally, SCiMS can also serve as a valuable quality control (QC) step in metagenomic analyses even when sex information has been collected, identifying potential sample swaps or metadata inconsistencies.

However, SCiMS has some limitations and areas that could be further developed or benchmarked. Its accuracy depends on the presence of reads mapping to host sex chromosomes. While it shows greater performance than existing tools at low host read depths, it will still report inconclusive sex calls for samples with extremely low read depths or where host sex chromosome reads are absent. We benchmarked SCiMS performance using a robust posterior probability threshold of 0.8, which was selected to balance precision and recall. However, users wishing for even higher precision have the option to increase that threshold. Additionally, SCiMS is expected to be most effective in organisms with well-characterized reference genomes, potentially limiting its applicability to species without high-quality genomic resources. The performance of SCiMS on lower quality genomes has yet to be benchmarked and would be an open area of interest to those who intend to use it for non-model organisms. Finally, SCiMS is currently tailored specifically for XY and ZW sex determination systems. This currently prohibits its use in organisms with other sex determination mechanisms, such as those with homogametic, polygenetic, or environmental sex determination systems. Regardless, SCiMS offers a novel, accurate new framework for calling sex from host-derived metagenomic samples.

Importantly, the purpose of SCiMS is to infer specifically the chromosomal sex of host organisms with heterogametic sex chromosomes (XY or ZW). Gender identity cannot be inferred using SCiMS, as gender is a social construct not inherently tied to biological sex [24]. Additionally, SCiMS may have limitations in accurately identifying sex in individuals with sex chromosome aneuploidies, where individuals are born with more or fewer than two sex chromosomes. These variations include conditions like Klinefelter syndrome (XXY), Turner syndrome (X), or XYY syndrome (XYY) [25]. Finally, SCiMS may not recapitulate the reported sex of individuals whose sex-related biological characteristics do not align with binary definitions of male or female. Therefore, while SCiMS offers a novel bioinformatic approach for predicting chromosomal sex, it is essential to acknowledge its inferences are limited to binary sex chromosome models and do not capture biological or social dimensions of sex and gender beyond chromosomal inference.

Finally, researchers should consider the ethical implications of applying SCiMS to their own or publicly available datasets, particularly when analyzing human-derived data. Sex is widely considered sensitive personal data and inadvertent disclosure of this information can have privacy implications [26, 27]. Following recommendations from a 2022 U.S. Department of Health and Human Services-sponsored report, sex should only be collected when sex is relevant to a study’s scientific aims [24]. Furthermore, host reads derived from metagenomic samples may contain other sensitive genetic information beyond sex that can reveal genetic ancestry or even individual genetic variants with potential clinical relevance [8]. While it is considered good practice to filter out host-derived reads from metagenomic datasets prior to deposition in publicly available repositories, host reads can still be present if insufficient filtering is performed [28]. Researchers are encouraged to ensure datasets they collect are deposited either in controlled access repositories (such as dbGaP) or are thoroughly filtered before deposition. Beyond technical safeguards, ethical use of inferred host metadata depends on study context and participant consent. As with any inference of sensitive host metadata, the application of SCiMS to human-derived metagenomic data should be consistent with existing ethical approvals and informed consent governing the original data collection, enabling responsible use of inferred sex information in microbiome research.

In conclusion, we developed SCiMS to fill the critical gap in accurately determining host sex from metagenomic data, particularly at low host read coverages. Our findings demonstrate that SCiMS not only outperforms existing methods in these challenging scenarios, but also offers potential applications in sample quality control processes. While SCiMS has limitations, particularly regarding the quality of the reference genome and the sex chromosome content of the sample, it represents a valuable tool that helps scientists identify the sex of each sample. This allows them to incorporate sex as a metadata variable in their analysis or confirm metadata integrity, ultimately making microbiome research more accurate and reliable.

## Conclusions

In this study, we presented SCiMS, a robust and scalable probabilistic tool designed to predict host sex from metagenomic sequencing data. By utilizing sex-chromosome, autosomal chromosome read density, and a Bayesian classification model, SCiMS overcomes the limitation of current genomic tools to accurately call host sex in low-host-biomass samples. Our results demonstrate that SCiMS maintains high accuracy even at ultra-low host coverage, requiring as few as 450 host reads. Beyond human studies, we have shown that SCiMS effectively generalizes across diverse biological systems, including murine (XY) and avian (ZW) models. By providing a reliable method to recover or verify sex metadata, SCiMS can be incorporated into downstream statistical models, thereby enhancing the reproducibility and rigor of metagenomic studies.

## Supplementary Material

Supplement 1

## Figures and Tables

**Table 1.** Metagenomic datasets.

Dataset	Species (assembly)	Samples (n)	Average host read count (reads)	Accession / Citation
Human Microbiome Project	*Homo sapiens* (GRCh38.p14)	1339*	1.2 10^5^ ± 2.6 10^5^	dbGaP phs000228 [12]
Mouse gut	*Mus musculus* (GRCm39)	111^†^	32.5 10^5^ ± 33.8 10^5^	NCBI SRA PRJNA517295 [16]
Chicken cecum	*Gallus gallus* (GRCg7b; GCA 027557775.1)	94^‡^	1.0 10^5^ ± 3.69 10^5^	ENA PRJEB34567 [17], PRJNA658643 [18], & PRJNA947933 [19]

* 718 males and 621 females

† 23 males and 88 females

‡ 22 males and 72 females.

## Data Availability

The HMP dataset was downloaded from dbGaP Study Accession: phs000228. The mouse cecal dataset is available at NCBI SRA project number PRJNA517295. The chicken cecal datasets are available at the ENA project number PRJEB34567 and NCBI SRA project numbers PRJNA658643 and PRJNA947933. All source code, Snakemake pipelines, Conda environment files and parameter settings to recreate the analyses in this paper are available at https://github.com/davenport-lab/SCiMS-paper. The SCiMS tool is available at https://github.com/davenport-lab/SCiMS.

## Abbreviations

FN: False Negatives
FP: False Positives
HMP: Human Microbiome Project
KDE: Kernel Density Estimation
PAR: Pseudoautosomal Region
QC: Quality Control
SCiMS: Sex Calling in Metagenomic Samples
TP: True Positives
