# Supplementary material for: SCiMS: Sex Calling in Metagenomic Sequences
Source: bioRxiv. 2026 Feb 18:2026.02.17.705110. Preprint. [Version 1] doi: 10.64898/2026.02.17.705110 (PMC12934929; doi:10.64898/2026.02.17.705110)
Supplement: Supplement 1 [file media-1.docx]

Supplementary Figures and Tables
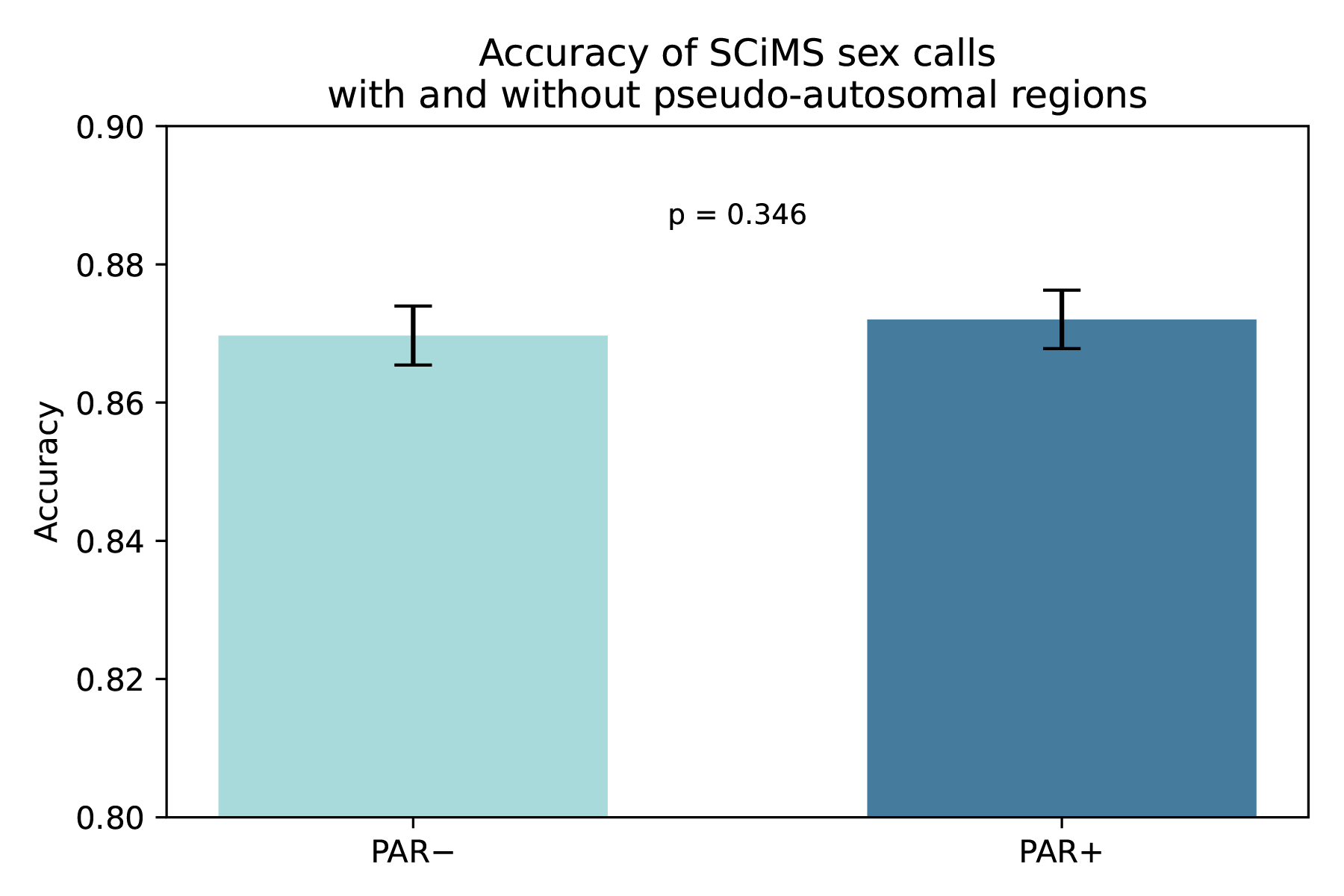


**Supplementary Fig. 1: SCiMS performance is consistent with the inclusion of Pseudoautosomal Regions (PARs).** Comparison of overall classification accuracy on simulated data when PARs are included (unmasked) versus excluded (masked) from the alignment. While filtering PARs is recommended to avoid coverage bias, SCiMS maintains high accuracy even when these regions are not explicitly removed, demonstrating reliability in cases where PAR annotations are unavailable.


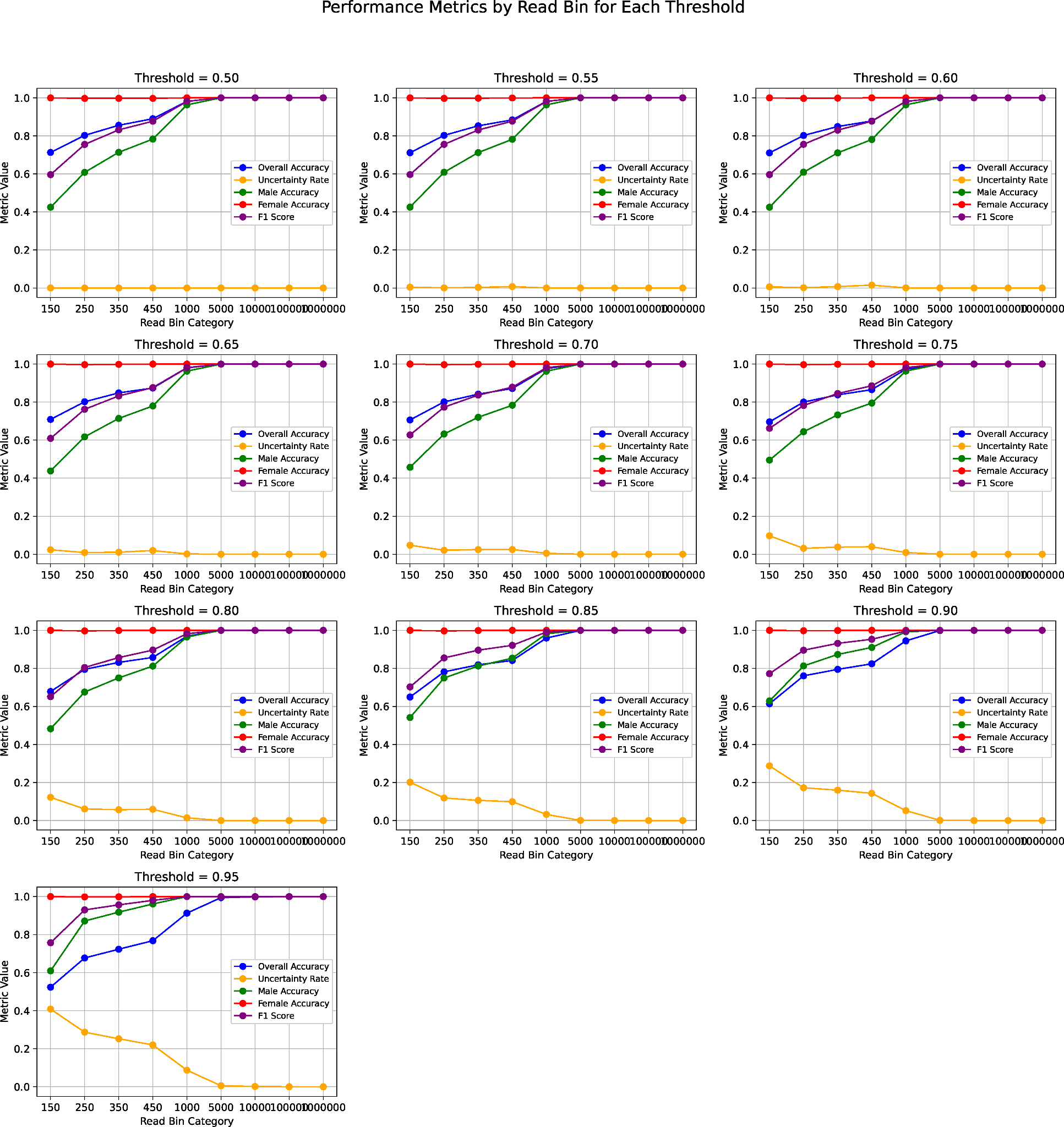


**Supplementary Fig. 2. Optimization of the Bayesian posterior probability threshold.** Evaluation of SCiMS performance metrics (Precision, Recall, Accuracy, and Uncertainty Rate) across a wide range of posterior probability thresholds from 0.50 to 0.99. A threshold of 0.80 was selected as the default parameter, as it provides the optimal balance between minimizing false positives (high precision) and maximizing data recovery (high recall) across varying sequencing depths.

**Supplementary Fig. 3. Comparative performance of SCiMS and existing tools on HMP dataset.** Classification outcomes for SCiMS, BeXY, Rx, and Ry applied to 1,399 samples from the Human Microbiome Project. Stacked bars represent the proportion of samples classified as Correct (cyan), Incorrect (orange), or Uncertain (grey). While Ry achieves the highest overall accuracy in this dataset, SCiMS demonstrates a good balance between precision and recall. Unlike BeXY and Rx, which exhibit rate of misclassification (orange, BeXY) or extreme uncertainty (grey, Rx), SCiMS successfully recovers most of these samples with high precision, offering a more reliable probabilistic framework than Ry.
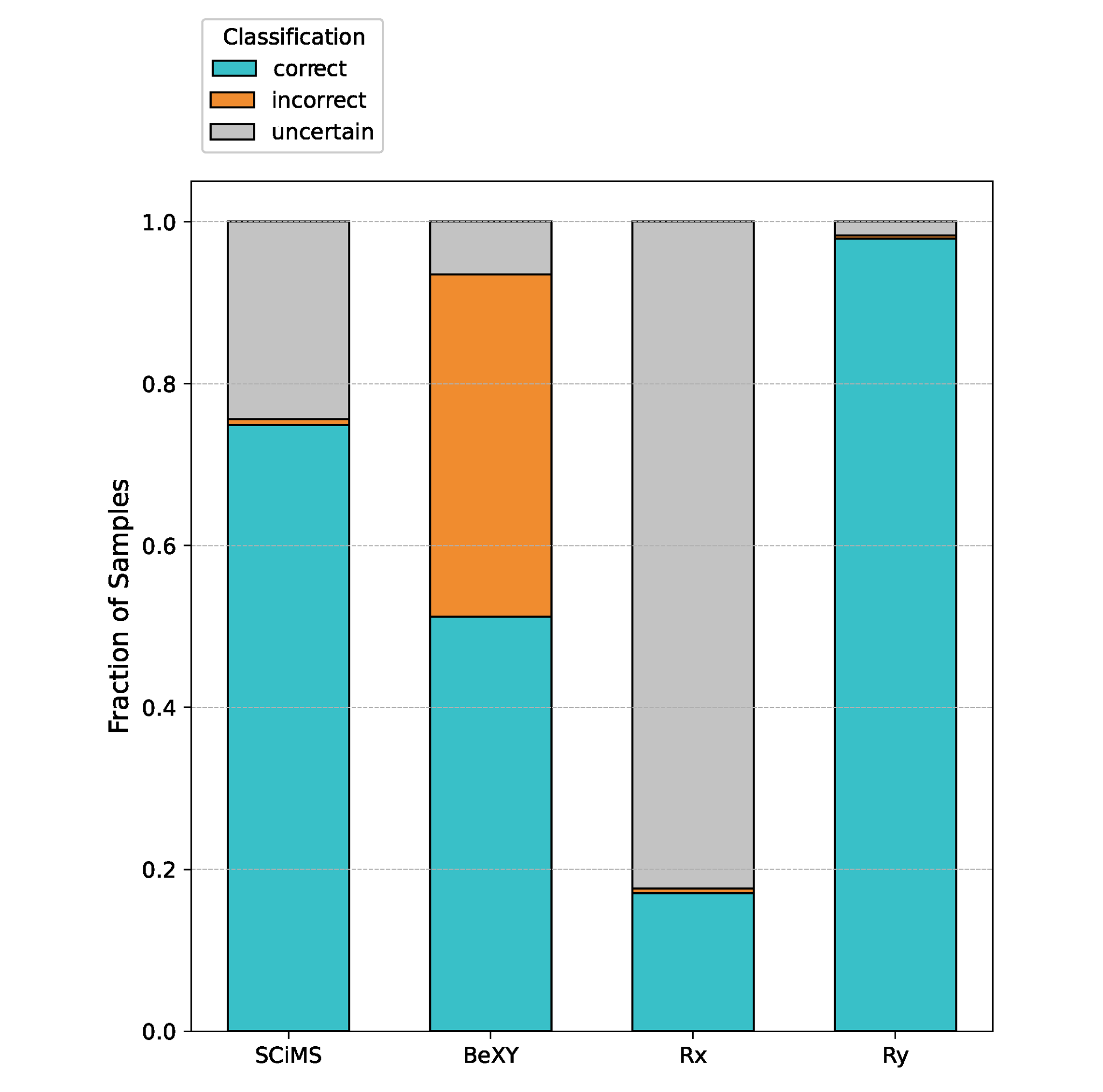


**Supplementary Fig. 4. SCiMS’ classification accuracy improves with host read counts across all body sites in the HMP dataset.** The relationship between host read depth and classification stratified by body site. Solid lines represent the mean accuracy, and shaded regions indicate the 95% confidence interval. SCiMS performance correlates strongly with sequencing depth.
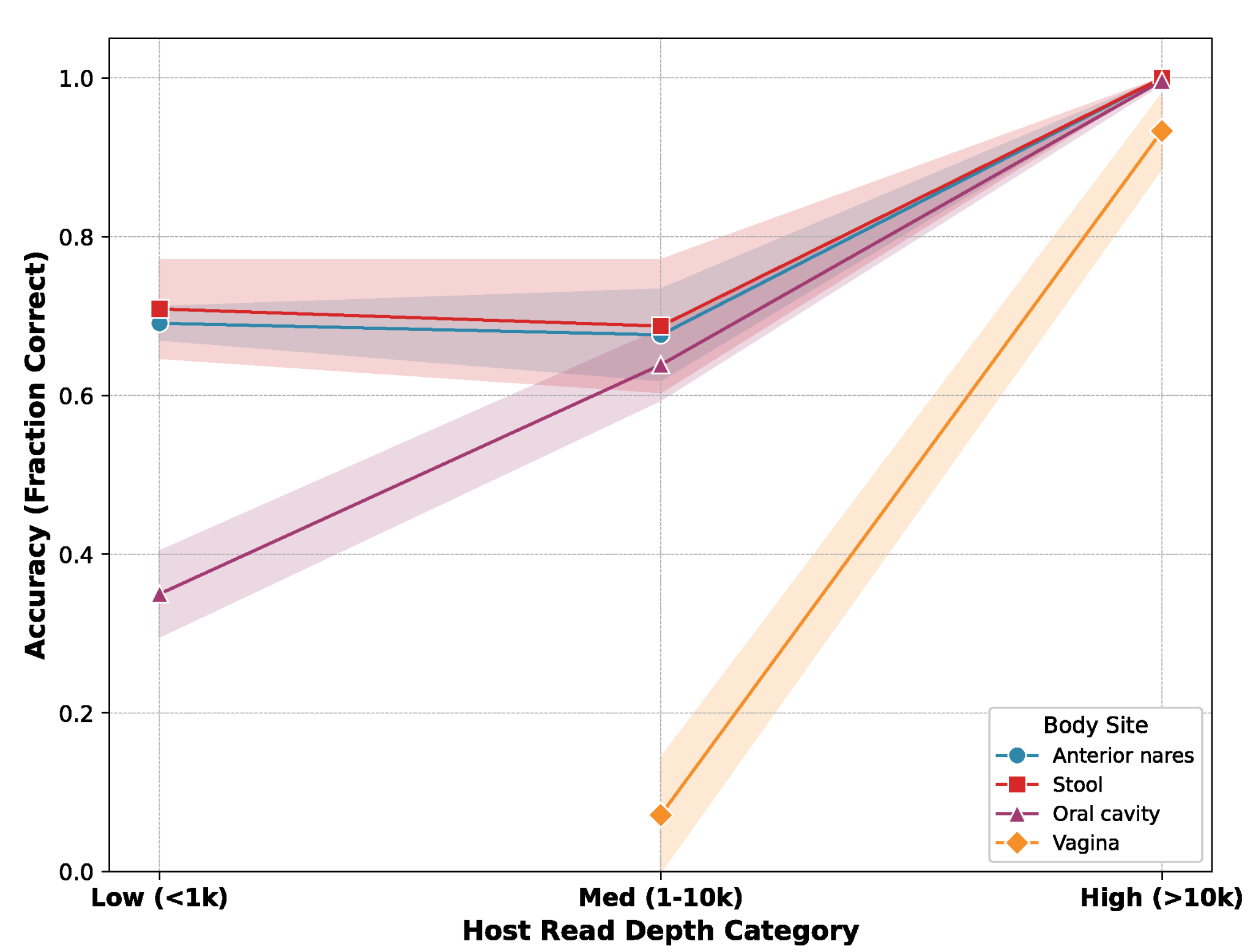


**Supplementary Table 1. Classification accuracy of sex inference tools on simulated human data across varying sequencing depths.** Accuracy values (proportion of correctly classified samples) for SCiMS, BeXY, Rx, and Ry at host read counts ranging from 150 to 1,000,000 reads. Each depth includes 2,000 simulated replicates (1,000 males and 1,000 females)

| Read depth | SCiMS | BeXY | Rx | Ry |
| --- | --- | --- | --- | --- |
| 150 | 0.6784 | 0.3892 | 0.3818 | 0.5125 |
| 250 | 0.7948 | 0.6148 | 0.5323 | 0.5073 |
| 350 | 0.8315 | 0.7373 | 0.562 | 0.5078 |
| 450 | 0.8575 | 0.8395 | 0.6105 | 0.511 |
| 1000 | 0.969 | 0.9835 | 0.7554 | 0.5213 |
| 5000 | 1 | 1 | 0.979 | 0.6615 |
| 10000 | 1 | 1 | 0.998 | 0.817 |
| 100000 | 1 | 1 | 1 | 1 |
| 1000000 | 1 | 1 | 1 | 1 |

**Supplementary Table 2. Sex-stratified performance metrics on simulated data.** Precision, Recall, and F1-scores for males and females classification tasks on simulated dataset. Metrics are aggregated across all simulated read depths to summarize overall performance.

| Class | Precision | Recall | F1-score | n | Method |
| --- | --- | --- | --- | --- | --- |
| male | 0.8150 | 0.896 | ​​0.854 | 11986 | SCiMS |
| female | 0.999 | 0.995 | 0.997 | 11997 | SCiMS |
| male | 0.994 | 0.800 | 0.887 | 11986 | BeXY |
| female | 0.998 | 0.756 | 0.860 | 11997 | BeXY |
| male | 0.976 | 0.666 | 0.791 | 11986 | Rx |
| female | 0.986 | 0.735 | 0.843 | 11997 | Rx |
| male | 0.538 | 0.339 | 0.416 | 11986 | Ry |
| female | 1.000 | 0.997 | 0.998 | 11997 | Ry |

**Supplementary Table 4. Comparative accuracy of sex inference methods across human body sites and non-human species**. Classification accuracy (percentage of correct calls) for SCiMS, BeXY, Rx, and Ry. Human samples are stratified by body sites to highlight performance in varying biomass contexts. Mouse (*Mus musculus)* and Chicken (*Gallus gallus*) datasets demonstrate method generalization to other XY and ZW systems.

| Methods | Human anterior nares | Human oral | Human vaginal | Human stool | Mouse cecal | Chicken cecal |
| --- | --- | --- | --- | --- | --- | --- |
| SCiMS | 74.98 | 80.53 | 65.9 | 72.04 | 100 | 69.1 |
| BeXY | 51.2 | 30.83 | 68.18 | 59.14 | 96.4 | 24.47 |
| Rx | 17.07 | 44.02 | 18.18 | 38.71 | 85.59 | 5.32 |
| Ry | 97.88 | 98.17 | 95.54 | 59.14 | 79.28 | 20.21 |

**Supplementary Table 6. Detailed performance metrics of SCiMS for the HMP dataset.** Precision, Recall, and F1-scores for each method applied to the full HMP cohort (n=1,339). Metrics are reported separately for male and female samples to highlight sex-specific biases in classification performance.

| Class | Precision | Recall | F1-score | n | Method |
| --- | --- | --- | --- | --- | --- |
| male | 0.98036466 | 0.98589563 | 0.98312236 | 709 | SCiMS |
| female | 0.97014925 | 0.95870206 | 0.96439169 | 339 | SCiMS |
| male | 0.54617834 | 0.53846154 | 0.54229249 | 637 | BeXY |
| female | 0.46642468 | 0.47416974 | 0.47026532 | 542 | BeXY |
| male | 0.98130841 | 0.98130841 | 0.98130841 | 321 | Rx |
| female | 0.91780822 | 0.91780822 | 0.91780822 | 73 | Rx |
| male | 0.99705449 | 0.97691198 | 0.98688047 | 693 | Ry |
| female | 0.97394137 | 0.99666667 | 0.98517298 | 600 | Ry |

Supplementary method: Mathematical Framework of SCiMS

# 1. Overview

SCiMS is a probabilistic tool designed to infer host sex from metagenomic data. It uses a Bayesian classifier in combination with non-parametric Kernel Density Estimation (KDE). The model quantifies the posterior probability of a sample belonging to a specific sex class $S \in\{Male, Female\}$ given the observed coverage distribution of its sex chromosomes.

# 2. Coverage Ratios

For each sample, SCiMS extracts read mapping statistics from BAM files (via samtools idxstats) to calculate two primary features: Homogametic ratio ($R_{X}$ or $R_{Z}$) and Heterogametic ratio ($R_{Y}$ or $R_{W}$).

## 2.1 Homogametic Ratio ($R_{X}$ or $R_{Z}$)

The homogametic ratio represents the read density of the X (or Z) chromosome relative to the mean read density of the autosomes. The read density for any chromosome *c* is defined as:

$$D_{c}=\frac{number of reads}{length of chromosome} =\frac{N_{c}}{L_{c}}$$

We use read density rather than raw read counts to normalize for the variation in chromosome lengths.

$R_{X} = \frac{D_{X}}{{\frac{1}{|A|}\Sigma}_{a \in A} D_{a}}$

Where:

- $A$ is the set of all autosomal scaffolds.
- $D_{X}$ is the read density of chromosome X.
- $D_{a}$ is the read density of chromosome a, where $a \in A$.
- $|A$| is the total count of autosomal scaffolds used.

## 2.2 Heterogametic Ratio ($R_{Y}$ or $R_{W}$)

The heterogametic ratio represents the proportion of sex-chromosome-specific reads that originate from the Y (or W) chromosome. The raw fraction of reads mapping to Y is given by:

$R_{X} = \frac{N_{Y}}{N_{X}+ N_{Y}}$

Where:

- $N_{Y}$ is the number of reads mapping to the Y chromosome.
- $N_{X}$ is the number of reads mapping to the X chromosome.

# 3. Statistical Modeling via Kernel Density Estimation

To avoid assumptions about the parametric normality of the data, SCiMS generates the joint distribution of observed ratios $x = (R_{X}, R_{Y})$ using Gaussian Kernel Density Estimation (KDE). For each sex class$S \in\{Male, Female\}$, a probability density function $\hat{f}_{S}$ is learned from a training set of $n$ = 20,000 simulated samples. The KDE sums Gaussian kernels centered at each training data point $i$:
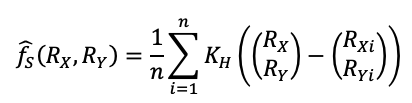


Where $n$ is the number of samples in the training dataset and $K_{H}$ is the Gaussian kernel with bandwidth matrix $H$ determined using Scott’s Rule default implemented in the scipy.stats package.

# 4. Bayesian Classification

SCiMS uses Bayes’ theorem to compute the posterior probability of each sex given the observed data $x$.

We define the prior probability of each sex as a uniform distribution, showing unbiased uncertainty of the prior knowledge to the sex of the sample:

$$P(Male) = P(Female) = 0.5$$

The likelihood of for each sample given a specific sex is taken directly from the KDE distributions:

$$P(x|Male) \approx\hat{f}_{Male}(R_{X} ,R_{Y})$$

$$P(x|Female) \approx\hat{f}_{Female}(R_{X} ,R_{Y})$$

The joint posterior probability for being male is computed as:

$$P(Male|x) \approx\frac{P(x|Male)P(Male)}{P(x|Male)P(Male) + P(x|Female)P(Female)}$$

$$P(Female|x) = 1-P(Male|x)$$

A sample is assigned a classification $\hat{S}$ based on a user-defined probability threshold $\tau$ (default $\tau$ = 0.80):

$\hat{S}$ = $Male$ if $P(Male|x) > \tau$

$\hat{S}$ = $Female$ if $P(Female|x) > \tau$

$\hat{S}$ = $Uncertain$ otherwise

Samples falling below the confidence threshold are flagged as “Uncertain” to prevent forced classification of low-quality or ambiguous data.
